# Effectiveness of Single Therapeutic Project in Adolescents With Attempted Suicide And/or Self-Harm

**DOI:** 10.1192/bjo.2022.242

**Published:** 2022-06-20

**Authors:** José Robson Samara Rodrigues de Almeida Junior, Yoichi Takaki Konno, Nágila Batista Lúcio Santos, Gabriel Silveira Parreira, Loíse Maria Tiozzo Cenedesi, Bianca Besteti Fernandes, Pedro Henrique Ferreira Carvalho

**Affiliations:** 1 FAMERP, Sao Jose do Rio Preto, Brazil; 2 CAPSi, Sao Jose do Rio Preto, Brazil

## Abstract

**Aims:**

Brazil is the eighth in number of suicides in the American continent, and 4th in the number of suicides in Latin America. From 2011–2016, there were 33,269 cases in women and 14,931 in men, with 24.1% and 17.2% in populations aged 10–19 years, respectively. Regarding self-harm, there were 116,113 cases (25.9%), and 60,098 cases (19.6%). And in both, the recurrence rate was high, with 25.3–33.1%. Properly employed, the Singular Therapeutic Project (STP) can prevent further events and even completed suicide. In this way, there is a reduction in stigma in this population and for family members, adequacy of mental health services, in addition to lower public spending on hospitalizations. We analysed the effectiveness of the STP in cases of suicide attempt and self-harm, in an outpatient setting, through the comparison of a group of patients with more adherence with the STP, in relation to the group with less adherence in a multidisciplinary context.

**Methods:**

It was a retrospective cohort, with adolescents aged 12 to 18 years, from the south of Sao Jose do Rio Preto (Brazil), during the period of 2015–2019, with follow-up for more than 3 months.

To analyze the behavior of numerical variables, descriptive statistics, boxplot plots and the specific test for Kolmogorov-Smirnov will be considered. Comparisons of continuous variables between two independent groups will be performed using Student's t test or Mann-Whitney test; comparisons of categorical variables with Pearson's chi-square test or Fisher's exact test.

**Results:**

The study sample consisted of 88 patients, 79.5% of whom were girls, 1.1% were illiterate, 34.2% were referred from the health unit, 29.5% from the hospital emergency. Patients with new episodes of suicide attempts accounted for 14.7% (1 attempt −10.2%, 2–1.1%, 3–3.4%), and self-harm 23.8% (1–12.5%, 2–2.3%, 4–4.5%, 5–2.3%, 6–1.1%, 7–1.1%).

There was no statistical significance between the group with adherence to STP and without adherence in relation to new suicide attempts (p = 1.0) and recurrence of self-harm (p = 0.309). In addition, both outcomes were not statistically significant with sociodemographic data, psychoactive substance use, and negative life events.

**Conclusion:**

The adolescents' adherence on STP was not associated in recurrence of suicide attempts and self-harm. We hypothesized that the sample size may have influenced the power of the statistical analysis. This pilot study is the first phase of the still ongoing study on all city.

